# Cardiac allograft vasculopathy: diagnosis, therapy, and prognosis

**DOI:** 10.3325/cmj.2014.55.562

**Published:** 2014-12

**Authors:** Boško Skorić, Maja Čikeš, Jana Ljubas Maček, Željko Baričević, Ivan Škorak, Hrvoje Gašparović, Bojan Biočina, Davor Miličić

**Affiliations:** 1 Department of Cardiovascular Diseases, University Hospital Center Zagreb, University of Zagreb School of Medicine, Zagreb, Croatia; 2 Department of Cardiac Surgery, University of Zagreb School of Medicine, University Hospital Center Zagreb, Zagreb, Croatia

## Abstract

Development of cardiac allograft vasculopathy represents the major determinant of long-term survival in patients after heart transplantation. Due to graft denervation, these patients seldom present with classic symptoms of angina pectoris, and the first clinical presentations are progressive heart failure or sudden cardiac death. Although coronary angiography remains the routine technique for coronary artery disease detection, it is not sensitive enough for screening purposes. This is especially the case in the first year after transplantation when diffuse and concentric vascular changes can be easily detected only by intravascular ultrasound. The treatment of the established vasculopathy is disappointing, so the primary effort should be directed toward early prevention and diagnosis. Due to diffuse vascular changes, revascularization procedures are restricted only to a relatively small proportion of patients with favorable coronary anatomy. Percutaneous coronary intervention is preferred over surgical revascularization since it leads to better acute results and patient survival. Although there is no proven long-term advantage of drug-eluting stents for the treatment of in-stent restenosis, they are preferred over bare-metal stents. Severe vasculopathy has a poor prognosis and the only definitive treatment is retransplantation. This article reviews the present knowledge on the pathogenesis, diagnosis, treatment, and prognosis of cardiac allograft vasculopathy.

The leading causes of death during the first three years after heart transplantation are nonspecific graft failure and infections. Nonspecific graft failure may be caused by chronic graft rejection, while acute graft rejection accounts for no more than 11% of mortality. The major determinants of patient survival after three years are malignancy and cardiac allograft vasculopathy (CAV), also known as transplant coronary artery disease or cardiac transplant vasculopathy. It is detected by coronary angiography in 8% of patients by year 1, 30% by year 5, and 50% by year 10 after transplantation (1). Due to graft denervation, CAV typically develops without the warning symptoms of angina pectoris and manifests with symptoms of graft failure, arrhythmias, or even sudden cardiac death. Patients may present with atypical symptoms such as exertional dyspnea, gastrointestinal distress, diaphoresis, or syncope. It is not unusual that CAV is diagnosed after an incidental finding of Q-waves on ECG or loss of contractile function on a routine echocardiographic exam (2). The vasculopathic lesions in the proximal coronary segments are more focal and eccentric, while the mid and distal coronary segments are affected in a more diffuse and concentric pattern, with typical vessel pruning (Figure 1) (3). Proximal disease is donor-inherited and atherosclerotic in nature, while the mid- and distal disease is more immune-mediated and recipient-acquired. CAV is characterized by diffuse concentric intimal hyperplasia, ie, thickening of the epicardial arteries and concentric medial disease in the coronary microcirculation with the constriction of the external elastic membrane area and lumen loss (4). Unnoticed by coronary angiography, the most of the intimal thickening occurs during the first post-transplant year (5). The disease progression is often complicated by intracoronary thrombosis and subsequent, often silent, acute myocardial infarction (Figure 2) (6). Early mural thrombi primarily contain platelets, while succeeding thrombi are more organized, usually occlusive and primarily consist of fibrin (7). Development and progression of CAV in transplant patients is strongly associated with enhanced platelet activation, although no evidence supports the benefit from aspirin therapy in these patients (8,9).

**Figure 1:**
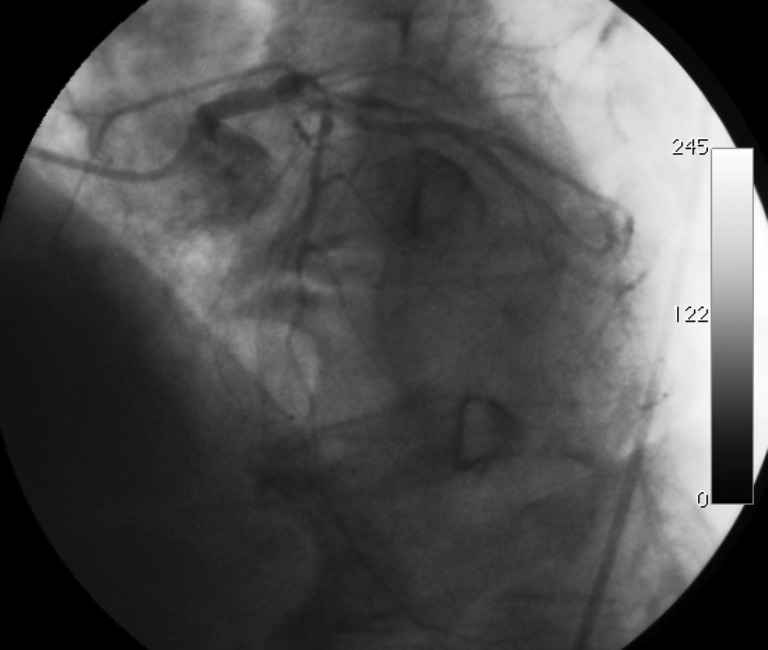
Diffuse stenosis of the left anterior descending artery and distal pruning of left circumflex artery in a patient 6 years after heart transplantation.

**Figure 2:**
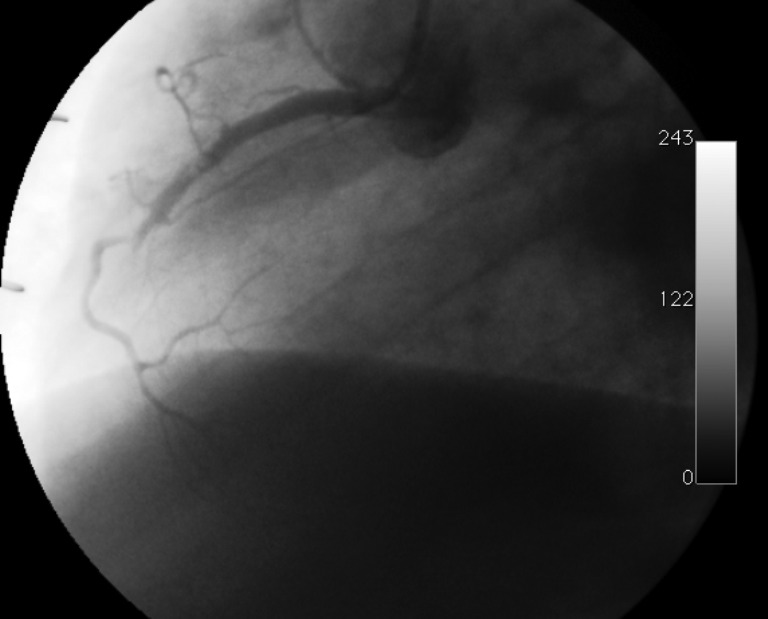
Acute thrombotic occlusion of the right coronary artery manifested as ventricular fibrillation and cardiac arrest in a patient 2 years after heart transplantation.

To create uniform definition and enable staging of transplant vasculopathy, the International Society of Heart and Lung Transplantation (ISHLT) proposed a grading system based on the combination of angiographic finding and graft function defined either by ultrasound or invasive hemodynamic measurement (10): CAV_0_ (no detectable angiographic lesions) = no vasculopathy; CAV_1_ (mild disease) = left main stenosis <50% or primary vessel stenosis <70% (including right coronary artery – RCA) or any branch stenosis <70%, without graft dysfunction; CAV_2_ (moderate disease) = left main stenosis <50% or single primary vessel stenosis >70% or isolated branch stenosis >70% in 2 systems, without graft dysfunction; CAV_3_ (severe disease) = left main stenosis ≥50% or stenosis >70% in two or more primary vessels or isolated branch stenosis >70% in three systems; or ISHLT CAV_1_ or ISHLT CAV_2_ with signs of graft dysfunction. Allograft dysfunction is defined as left ventricular ejection fraction ≤45% or evidence of significant restrictive pathology either on echocardiographic exam (E/A ratio >2, isovolumetric relaxation time <60 ms, deceleration time <150 ms) or right heart catheterization (right atrial pressure >12 mm Hg, pulmonary capillary wedge pressure >25 mm Hg, cardiac index <2 L/min/m^2^). In this nomenclature, primary vessels stand for the proximal and middle thirds of the left anterior descending artery, the left circumflex, the ramus intermedius, and the dominant or co-dominant RCA. Branch vessels denote distal thirds of the primary vessels or large septal, diagonal, and obtuse marginal branch or any portion of a non-dominant RCA (10). CAV is present in 44% of our patients at 5 years after transplantation with the proportion of CAV_1_, CAV_2,_ and CAV_3_ of 50%, 32%, and 18%, respectively.

## Pathogenesis

CAV development is related to numerous immunologic and non-immunologic factors. The immunologic factors seem to be of crucial importance since vasculopathy develops in the donor's rather than in the recipient's vessels. The role of inflammation is congruent with the observation that the increased C-reactive protein (CRP) is a strong predictor of CAV development after heart transplantation (11). Multiple immunologic mechanisms triggering coronary vessel injury are initialized after the host’s immune system recognizes non-self histocompatibility antigens (HLA) of the transplanted heart in the process of allorecognition. This is followed by T-cell activation, formation of donor specific antibodies, endothelial cell activation, and altered cytokine expression (12). Recipients who develop CAV show a greater incidence of HLA mismatching, while the HLA-DR mismatching best predicts an adverse outcome (13,14). The majority of trials found the risk of CAV to be related to the number of moderate and severe cellular rejection episodes, as well as to the total rejection score, which represents the summary of overall rejection (15-17). Antibody-mediated rejection, also known as humoral or vascular rejection, is characterized by anti-HLA and anti-endothelial antibodies formation, and it is also related to CAV development (18,19). The presence of anti-endothelial antibodies correlates strongly with the progression of vasculopathy (72% vs 5% in patients without antibodies) and the presence of anti-HLA antibodies with considerably lower 4-year survival (90% vs 38%) (20,21).

The graft endothelium represents the borderline between the donor’s and recipient’s immune systems and plays a critical role in the pathophysiology of CAV. The increased expression of major histocompatibility complex (MHC) class I alloantigens on coronary endothelial cells of the transplant heart are directly recognized by CD8+ T-cells, resulting in the secretion of cytokines with further activation of endothelial cells. The activated endothelial cells express increased levels of MHC class II antigens, which later activate CD4+ T-cells. This can explain the predominance of CD8+ T-cells in the early vasculopathic lesions and the increased proportion of CD4+ T-cells in the advanced stage of the disease (22). Endothelial activation is the initial step of immune response propagation, followed by recruitment of pro-inflammatory cells from the vessel wall. This is accompanied by marked expression of the intercellular cell adhesion molecule-1 (ICAM-1), vascular cell adhesion molecule-1 (VCAM-1), and P-selectin on the surface of allograft endothelial cells – a phenomenon also seen in the acute cellular rejection (23-25). In fact, endothelial activation during the first months following heart transplantation predicts future graft vasculopathy (26). Increased serum level of soluble ICAM-1 correlates with the level of endothelial ICAM-1 in the biopsy specimens and predicts both CAV development and graft failure (27). Endothelial activation results in endothelial dysfunction, which may be detected as abnormal coronary vasomotor response to acetylcholine or adenosine on angiography (28).

Multiple cytokines with proliferative effects on coronary smooth muscle cells have been detected within vasculopathic lesions: interleukin-1 (IL-1), interleukin-6 (IL-6), tumor necrosis factor-alpha (TNF-α), platelet derived growth factor, insulin-like growth factor-I, macrophage chemoattractant protein-1 (MCP-1), fibroblastic growth factor, vascular endothelial growth factor, transforming growth factor-alpha, and TGF-beta-1 (29-34). Interferon-gamma (IFN-gamma) is secreted by T-cells and performs a variety of actions: activates macrophages, enhances the expression of MHC antigens and other components of the antigen presentation pathway as well as the adhesion molecules ICAM-1 and VCAM-1. The deficit of IFN-gamma was found to protect mice from transplant vasculopathy (35), while some genetic polymorphisms of TNF-α and IL-6 have been related to CAV development (36,37).

Non-immunologic factors associated with increased risk of CAV include age, sex, recipient body mass, diabetes mellitus, smoking, dyslipidemia, donor history of arterial hypertension, recipient history of previous coronary artery disease, cytomegalovirus (CMV) infections, defective fibrinolysis, polymorphism in angiotensin converting enzyme gene, early graft dysfunction due to preservation damage, ischemia-reperfusion injury, as well as the etiology of brain death of the donor (38). Non-immunologic factors cause endothelial dysfunction and damage, as well as promote the activation of the coagulation pathway. They also initiate nonspecific vascular inflammation, which induces the expression of endothelium-derived MHC antigens and promotes traveling of dendritic cells into the allograft, resulting in increased graft immunogenicity and recipient alloimmune response.

While older age and male sex of the donors are associated with higher risk, older age of the recipient is associated with lower risk for CAV (14,38,39). CAV severity correlates with the increase in total serum cholesterol, low-density lipoprotein (LDL)-cholesterol, oxidized LDL-cholesterol, and triglycerides, which are common metabolic abnormalities after heart transplantation due to use of corticosteroids and cyclosporine, and obesity (40,41). Lipid-lowering treatment with statins is one of the few therapeutic approaches able to reduce both the development and progression of allograft vasculopathy (42).

The relation between CAV development and CMV infection can be explained by stimulation of immune response in the vessel wall via induced expression of endothelial MHC antigens and direct endothelial damage resulting in dysregulation of the NO pathway, activation of coagulation cascade, cytokine secretion, and vascular remodeling with vessel lumen loss (43,44).

Glucose intolerance and insulin resistance are associated with higher likelihood of CAV and increased mortality (45,46). Metabolic syndrome, characterized by abdominal obesity, hypertension, diabetes, and dyslipidemia (hypertriglyceridemia and low HDL-cholesterol) is frequently observed in transplant patients. The combination of high CRP and insulin resistance, defined as triglyceride to HDL ratio >3.0, synergistically causes a 4-fold increase in CAV risk (47). Allograft vasculopathy is more likely to occur if either the recipient or the donor have the history of coronary artery disease (39,48). The risk for CAV may also be increased by impaired fibrinolytic system with procoagulant microcirculation and depletion of tissue-type plasminogen activator (tPA) from the arteriolar wall (49). Local tPA depletion may be due to genotype-specific overexpression of plasminogen activator inhibitor-1 (PAI-1), with the donor 2/2 genotype associated with an increased risk of CAV (50,51). Graft vasculopathy is also related to the depletion of vascular antithrombin (52).

The paradoxical constriction of coronary arteries in response to acetylcholine is a sign of endothelial dysfunction and is predictive of vasculopathy (53,54), while the up-regulation of inducible nitric oxide synthase and increased expression of heat shock protein 27 seem to play protective roles (55,56). CAV development is also associated with the DD genotype of angiotensin converting enzyme gene polymorphism (57). The up-regulation of endothelin-1 in the coronary vessels contributes to vasculopathic changes by vasoconstriction, as well as by proliferation of intima and media (58). Endothelial injury may accompany myocardial damage that occurs with the brain death of the donor, suboptimal myocardial preservation, and ischemia-reperfusion graft injury following transplantation. This injury may be detected as early graft failure with high risk of future graft vasculopathy (59). The systemic activation of matrix metalloproteinase-2 is detected in donors after explosive brain death (ie, head trauma, intracerebral hemorrhage, gunshot wound), which is also related to CAV (60). Other factors that were associated with an increased risk of CAV include recipient obesity, recipient ischemic cardiomyopathy (vs non-ischemic cardiomyopathy), increased myocardial elastase activity, hepatitis C virus seropositivity, and increased blood levels of homocysteine or von Willebrand factor (38,61-64).

The complex interactions between the immunologic and non-immunologic factors cause endothelial damage with consequent endothelial activation and dysfunction. Endothelial activation stimulates expression of adhesion molecules and cytokine secretion, which further promote cell invasion, smooth muscle cells growth, and vascular matrix deposition, finally resulting in typical morphological vascular changes. Derangement of endothelial repair mechanism in transplant patients may also play a role. The level of circulating human endothelial progenitor cells (EPCs) was found to be significantly decreased in patients with CAV compared with matched patients without evidence of the disease. This may be explained by increased consumption of circulating pool of EPCs at the sites of graft endothelial injury. However, mobilization and survival of circulating EPCs was found to be suppressed by inflammation (65).

## Diagnosis

Due to the graft denervation, CAV will unlikely be diagnosed based on typical symptoms of angina pectoris. However, it should be suspected during routine echocardiographic examination if signs of graft dysfunction, such as regional wall abnormalities and/or restrictive diastolic dysfunction, are found. When it becomes symptomatic, CAV is usually at an advanced stage and myocardial damage is irreversible. For this reason, it is necessary to perform screening measures to improve early diagnosis.

Invasive screening methods include coronary angiography and intravascular ultrasound (IVUS). They cause some discomfort to the patient, involve the risk of renal damage from contrast agent application, and are resource consuming. Other methods include analysis of microvascular changes on routine endomyocardial biopsies and noninvasive methods like stress echocardiography, CT coronary angiography and measurement of serum biomarkers.

Coronary angiography is used to establish the diagnosis of CAV in most transplant centers. Depending on the center, the baseline angiography is commonly performed several weeks after the transplantation, while the following ones are performed annually or biannually. In our center, it is routinely performed biannually. However, coronary angiography is performed annually or even more frequently after the diagnosis of CAV has been established or if there are signs of new-onset graft dysfunction on echocardiographic exam. Coronary angiography can identify luminal narrowing as well as slow contrast filling of the distal coronary vessels, suggesting the involvement of small coronary vessels and microcirculation. As coronary spasm may mimic concentric vessel narrowing in vasculopathy, intracoronary nitroglycerine should be applied before contrast injection in order to avoid false CAV diagnosis. Although angiography remains the routine technique for coronary artery disease detection, it is not sensitive enough for CAV detection due to diffuse, longitudinal, and concentric vascular changes. The positive predictive power of coronary angiography compared to IVUS as the gold standard is only 44% (66). Consequently, transplant vasculopathy often remains angiographically undiagnosed at the early stage. Moreover, there is notable interobserver variation of angiographic diagnosis and the experience in this field of invasive cardiology is needed (67). Additionally, angiography may help to diagnose CAV by the measurement of TIMI (thrombolysis in myocardial infarction) frame count, ie, the number of cine frames required for the contrast to reach distal coronary segments. The increase in TIMI frame count was observed in patients who eventually developed angiographically confirmed CAV after five years of follow-up, although conflicting results were observed when TIMI frame count was correlated with IVUS finding (68). Coronary flow reserve (CFR) measures the increase in coronary blood flow in response to a vasodilator such as adenosine. The reduction of CFR may indicate the development of graft vasculopathy before angiographic changes occur (69).

Unlike angiography, IVUS can detect the abnormal concentric intimal thickening and is the most sensitive tool for CAV detection. The most rapid rate of intimal thickening occurs during the first posttransplant year (70). An increase in maximal intimal thickness (MIT) for ≥0.5 mm during that period indicates rapidly progressive CAV (71,72). Because of high positive predictive value, IVUS has been used as surrogate end-point in large multicenter clinical studies of drugs for the prevention and treatment of CAV. Disadvantages of IVUS include additional procedural risk, more time consumption, limited availability, and increased costs. However, IVUS is of crucial clinical importance in patients with unexplained graft failure, ie, those with normal coronary angiography and no detectable rejection.

Noninvasive tests include conventional electrocardiogram stress test, dobutamine stress echocardiography, coronary flow reserve (CFR) assessment by contrast-enhanced echocardiography, stress myocardial perfusion scintigraphy, positron emission tomography (PET) scanning, computed tomography (CT) angiography as well as serum biomarkers testing. These studies are less sensitive, but as they are not invasive and are generally less expensive, may serve as adjunctive methods of screening for myocardial ischemia (73). The best-validated noninvasive method is dobutamine stress echocardiography, with the sensitivity of 85% as compared to angiography and IVUS. Furthermore, its negative predictive value is outstanding, ie, patients with normal stress study were shown to be free of adverse events (74,75). Noninvasive screening is used for low-risk transplant patients, ie, those with previously normal angiography, no signs of graft dysfunction, and a time span of more then five years after transplantation. It is also used in patients with advanced renal failure when it is desirable to avoid the administration of contrast media. However, symptomatic patients or those with noninvasive tests results suggestive of CAV should be referred to coronary angiography or/and IVUS.

Contrast enhanced CFR has been evaluated by clinical studies as a method of screening, but it requires high-quality imaging that is often impossible to perform due to unfavorable acoustic window in transplanted patients (76). Although CT coronary angiography has a sensitivity between 85%-100%, it suffers from many drawbacks such as poor image quality, tachycardia, which is typically present in heart transplant patients, high burden of radiation, nephrotoxicity since a greater amount of contrast is required than in conventional angiography, and the problem of small vessel disease detection (77,78). CT coronary angiography is at present not recommended for routine CAV monitoring.

Biomarkers like CRP or N-terminal pro-brain natriuretic peptide (NT-proBNP) were predictive of mortality but not of CAV development (79). Serum NT-proBNP level <800 pg/mL was associated with better survival after transplantation (80). Higher serum level of von Willebrand factor was a predictor of allograft vasculopathy (64).

Immunohistochemistry evidence of endothelial activation in the arteries and arterioles of endomyocardial specimens taken during the first three months after transplantation precede morphological changes in vasculopathy (26). Stenotic changes of the coronary microcirculation in the biopsy sample may also accompany epicardial vasculopathy (81,82). The sensitivity of this method is low, but the positive finding highly predicts both CAV and death (83).

## Prognosis

CAV is a slowly progressive disease, though it may progress rapidly and result in graft failure, arrhythmias, or sudden cardiac death (SCD). Because of heart denervation, graft ischemia usually progresses without classic symptoms and SCD may be the first manifestation of vasculopathy. SCD may be prevented by cardioverter defibrillator implantation, though not entirely, as some of these deaths result from instantaneous and fatal heart failure (84). Significant prognostic factors for SCD are the presence of >25% coronary stenosis on angiography or the worsening of TIMI frame count after the first year of follow-up (85,86). An increase in maximal intimal thickness (MIT) on IVUS for ≥0.5 mm indicates rapidly progressive CAV and is associated with more deaths and/or graft losses at 5-year follow-up (20.8% vs 5.9% in patients with <0.5 mm) (71). The combined end-point of death and myocardial infarction was reached by 51% of patients with MIT increase of ≥0.5 mm vs 16% in patients with <0.5 mm MIT increase (72). The majority of epidemiological reports on the prevalence of CAV did not take into consideration the severity of this disease, which carries different prognostic risk. Costanzo et al (87) reported 42% CAV prevalence in the overall transplant population after 5 years. However, only 7% of patients had severe CAV defined as left main stenosis >70% or >70% stenosis in two or more primary coronary arteries or branch stenosis for >70% in all three coronary systems. Notably, the adverse outcome, including death and retransplantation, occurred in only 7% of the total number of CAV patients and even in two-thirds of patients with severe vasculopathy (87). It seems that late-onset graft vasculopathy is an unusual disorder and that its incidence is significantly lower in patients who survived more than ten years after transplantation (88). Despite the lack of substantial progress in CAV treatment, there was an increase in the survival of patients who were transplanted between 2001 and 2007 compared to those transplanted between 1994 and 2000 (89).

## Prevention

Because CAV has substantially poor prognosis when it becomes symptomatic, the primary effort is directed to early prevention, detection, and initiation of treatment. Only two types of drugs were found to provide significant protection from CAV: statins and mammalian target of rapamycin (mTOR) inhibitors.

Statins are considered mandatory in transplant patients because they improve patient survival, reduce both the incidence and severity of vasculopathy, as well as the incidence of graft rejection (90). They are typically instituted by the end of the first week or during the second week after heart transplantation. Pravastatin is started with 20 mg/d and then increased to 40 mg/d if tolerated (42). Simvastatin is started with 5 mg/d and increased to 10 and 20 mg/d (91). The early initiation of statins is of utmost importance, as their later introduction in transplant patients did not have a favorable effect on graft prognosis although it reduced serum cholesterol level (92). The favorable effects of statins, besides lowering plasma lipids, may be explained with anti-inflammatory activity, cytokine suppression, and improvement of endothelial function (93,94). In our center we also use fluvastatin, atorvastatin, or rosuvastatin. Special caution must be taken to avoid rhabdomyolysis, as most statins use CYP3A4 pathway that is already inhibited by calcineurin inhibitors (CNI). The risk is lower with fluvastatin as it is metabolized by CYP2C9, and with pravastatin, as it is excreted largely unchanged (95).

Sirolimus (SRL) and everolimus (EVL), also known as mTOR or proliferation signal inhibitors (PSI), inhibit T-cell and B-cell proliferation driven mainly by interleukins. Eisen et al showed superiority of EVL over azathioprine for the prevention of CAV in de novo heart transplant patients already on cyclosporine and corticosteroid therapy (96). After 12 months, the patients on EVL had significantly slower progression of MIT as determined by IVUS. EVL was associated with lower incidence of graft rejection and CMV infection, but also with more frequent renal failure, bacterial infections, dyslipidemia, and thrombocytopenia. The beneficial effect of EVL on MIT was maintained for 2 years of follow-up. Moreover, this treatment was associated with significantly fewer CAV-related events and revascularization during 4 years of follow-up (97).

SRL in de novo heart transplant patients treated with cyclosporine and corticosteroids reduced both MIT and ≥3A rejection grade in comparison to azathioprine (98). Disadvantages of SRL included renal insufficiency, abnormal wound healing, epistaxis, hyperlipidemia, anemia, and thrombocytopenia. In the early postoperative phase, SRL has been avoided due to the problem of slow wound healing in renal transplantation trials. Since the cause of renal function deterioration seems to lie in combination of mTOR inhibitors and calcineurin-inhibitor, a solution would be to reduce calcineurin-inhibitor concentrations (99). Mycophenolate mofetil (MMF) in higher dosage (2 × 1.5 g/d) reduced minimal intimal thickness on IVUS in comparison to azathioprine during 3 years of follow-up (0.06 vs 0.13 mm with azathioprine, *P* = 0.056). It also showed significantly lower incidence of retransplantation or death (100).

Diltiazem was shown to have benefits in a trial from the pre-statin era, which is not considered very strong evidence, especially since this trial used quantitative coronary angiography rather than IVUS (101). A later study in patients treated with statins found no benefits of diltiazem (102). Fang et al showed a favorable effect of antioxidant therapy during the first two years after transplantation in patients already on statins. Vitamin C (2 × 500 mg/d) and vitamin E (2 × 400 IU/d) reduced the progression of vasculopathy measured by IVUS and defined as the change in average intimal index (plaque area divided by vessel area) after one-year of follow-up (103). Improvement in endothelial protection by stimulation of the nitric-oxide pathway with oral l-arginine may reduce vascular oxidant stress in the graft, reverse endothelial dysfunction, and decrease blood pressure after transplantation, which may help to prevent vasculopathy (104,105). Aggressive CMV prophylaxis in all and not only in high-risk transplantation recipients correlated with less intimal thickening on IVUS (106). Interestingly, there is no evidence for benefit from standard antiplatelet therapy in heart transplant patients. This may be explained with high on-treatment platelet reactivity, ie, aspirin resistance after transplantation (9).

## Treatment

The treatment of posttransplant vasculopathy is not very promising and it primarily modifies immunosuppressive regimens. Selected patients may be treated with percutaneous coronary interventions (PCI), while only few patients are candidates for coronary artery bypass grafting (CABG). Revascularization procedures are associated with poor long-term results and are considered palliative due to the diffuse and progressive nature of vascular changes. Heart failure medications should be introduced with the signs of graft failure, while in patients with severe disease, the only option remains to be retransplantation.

Short-term enhancement of immunosuppressive therapy may slow down the progression of CAV (three days of methylprednisolone plus antithymocite globulin). This effect is more pronounced if applied early, ie, within the first posttransplant year. However, due to significant risk of infections and malignant diseases, this approach is not adopted in routine practice (107).

There is evidence that both EVL and SRL prevent CAV development, but established vasculopathy is treated only with SRL (107). A study in which antimetabolites (azathioprine or MMF) were replaced with SRL, while CNI and corticosteroids remained unchanged, showed a better outcome in patients with severe CAV – combined end-point of death, PCI, CABG, myocardial infarction and increase in semiquantitative catheterization score (108). However, MIT was measured with IVUS only in a minority of patients, in whom no difference was found (108).

Raichlin et al replaced CNI with SRL as a primary immunosuppressive drug in patients with vasculopathy and/or renal impairment (109). The secondary immunosuppressants (MMF or azathioprine) and corticosteroids were unchanged. The replacement resulted in stable plaque volume and plaque index (defined as plaque volume/vessel volume), while the CNI group exhibited further progression of vasculopathy (109). However, the Heart Save the Nephron trial was prematurely terminated as 4 of 7 patients in whom CNI drug was replaced with SRL 12 weeks after the heart transplantation experienced grade IIIA rejection (110). We recommend caution when SRL is used instead of CNI as a primary immunosuppressant during the early post-transplant phase before pathohistological evidence of durable rejection control in our patient is obtained.

**Figure 3:**
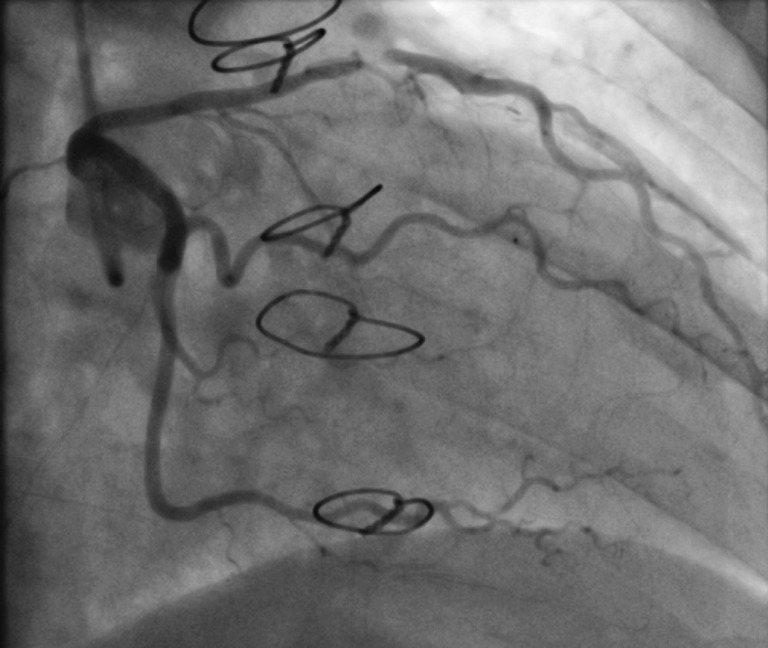
Subocclusive left anterior descending artery incidentally detected in an asymptomatic patient with anterior wall hypocontractility on routine echocardiographic exam 8 months after transplantation.

PCI is considered palliative because it does not influence the progressive nature of CAV. No studies tested the effect of PCI on the graft and patient survival. Optimal candidates for PCI are patients with focal and one vessel disease (Figure 3), who represent only a minority of patients with vasculopathy. Drawbacks of PCI in transplant patients include diffuse coronary disease, distal vessel pruning, common metabolic abnormalities (diabetes, dyslipidemia), the use of contrast agents in patients with nephrotoxic immunosuppressive therapy and preexistent renal failure, as well as high in-stent restenosis (ISR) rate. Although the immediate success rate of angioplasty was high (>92%), the restenosis rate was up to 55% during the next 15 months (111-115). Patients with calcified lesions might require rotablation, which has the periprocedural mortality of 18% (116). Compared to plain angioplasty, bare-metal stent (BMS) implantation resulted in higher primary procedural success rate and lower restenosis rate at three months (7% vs 39%) as well as at eight months (34% vs 71%) (117,118). However, in 33 patients with CAV there was no difference in restenosis rate (68% vs 69%) stenting after 5 years of follow-up (119). When compared to BMS, drug-eluting stents (DES) significantly reduced the restenosis rate in short and mid-term follow-up (≤19 months) (120-123). However, another study found no difference in restenosis rate between BMS or DES after 5 years of follow-up (124) but DES group had significantly higher prevalence of diabetes mellitus, longer time from heart transplantation to PCI, as well as a longer mean stent length. These factors might have influenced the susceptibility to stent restenosis and influenced the obtained results (124). SRL-eluting stents did not differ from paclitaxel-eluting stents with respect to binary restenosis, late lumen loss, or incidence of major adverse cardiac events after PCI (125). ISR is associated with progressive lumen loss in remote coronary lesions and, clinically more important, it represents a strong independent risk factor for future myocardial infarction and mortality in transplanted patients (126). The risk of ISR is increased by the presence of IgG antibodies to MHC class I antigen increases (127). A significant reduction of restenosis rate was achieved by treatment with MMF dose of ≥2 × 1.5 g/d (128).

Because of the diffuse nature of transplant vasculopathy and high perioperative mortality, surgical revascularization is an option in a very small number of patients with CAV. Coronary bypass grafting is associated with high perioperative mortality (36%) and only few patients with CAV are candidates for this type of revascularization (116).

## Other treatment modalities and future strategies

According to the present guidelines for SCD prevention in heart failure patients, implantable cardioverter defibrillators are used for the prevention of SCD in patients with severe CAV and reduced systolic function (129). However, there are no specific trials on this matter in the transplant population. For patients with terminal graft vasculopathy, the only definitive treatment is retransplantation. However, the survival at 5 years after heart retransplantation is <50%. The independent predictors of high mortality include the period of less than two years between two transplantations, the recipient age, intensive care treatment, mechanical ventilation, and the use of ventricular assist device as bridge-to-retransplantation (130). In practice, retransplantation is restricted only to highly selected patients, typically of young age.

As present CAV prevention and treatment modalities are very limited, there is a need for novel strategies. Alloimmune response may be prevented by induction of allograft tolerance by targeting local or circulating dendritic cells or by modulating T-cells co-stimulation pathways (131). Graft survival may be prolonged by cell therapy with autologous interleukin-10-engineered hematopoietic stem cells before transplantation, due to anti-inflammatory effect of this cytokine (132). Also, CAV in patients with deficient fibrinolysis or endothelial dysfunction may be prevented by induction of genes for tPA or endothelial nitric-oxide synthase by means of intracoronary gene transfer (133,134). Before more effective preventive and therapeutic strategies are developed, cardiac allograft vasculopathy will remain the most important limitation of long-term heart transplant survival.
